# Impact of energy intake on the association between protein intake and the prevalence of frailty in older Korean adults: The Korea National Health and Nutrition Examination Survey, 2014–2018

**DOI:** 10.1016/j.jnha.2025.100518

**Published:** 2025-02-19

**Authors:** Seokju Kang, Youri Jin, Yongsoon Park

**Affiliations:** a Department of Food and Nutrition, Hanyang University, 222 Wangsimni-ro, Seongdong-gu, Seoul 04763, Republic of Korea; b Department of Food and Nutrition Services, Hanyang University Seoul Hospital, 222-1 Wangsimni-ro, Seongdong-gu, Seoul 04763, Republic of Korea

**Keywords:** Older adults, Frailty, Protein, Energy, KNHANES

## Abstract

**Objectives:**

Findings regarding dietary protein and frailty are inconsistent. This inconsistency may be attributed to variations in energy intake adequacy. We hypothesized that the prevalence of frailty in older adults with sufficient energy intake is inversely associated with the intake of total, animal, and plant proteins; however, in those with deficient energy intake, this association may not be observed.

**Design, setting, participants, and measurements:**

This cross-sectional study included data of 5,768 adults aged ≥65 years from the Korea National Health and Nutrition Examination Survey 2014–2018. Frailty was assessed using a modified Cardiovascular Health Study frailty index. Protein intake was measured via a 24-h recall and analyzed using multivariable logistic regression.

**Results:**

In older adults with sufficient energy intake, frailty was inversely associated with the intake of total (odds ratio [OR], 0.67; 95% confidence interval [CI], 0.46–0.97; p = 0.032), animal (OR, 0.59; 95% CI, 0.43–0.82; p = 0.001), and plant proteins (OR, 0.68; 95% CI, 0.50–0.92; p = 0.043). In those with deficient energy intake, total and animal protein intake showed no significant associations with frailty, whereas plant protein intake was inversely associated with frailty.

**Conclusion:**

Adequate energy intake appears essential for the protective effects of total and animal protein against frailty. Higher plant protein intake appears to confer benefits regardless of energy intake, highlighting its potential role in frailty prevention.

## Highlights

• Frailty was inversely associated with protein intake under sufficient energy intake
• Plant protein intake reduced frailty risk regardless of energy intake levels
• Adequate energy intake was vital for protein’s protective effects against frailty

## 1 Introduction

Frailty is a common geriatric syndrome characterized by a state of increased vulnerability to stressors. Frailty increases the risk of adverse health outcomes, such as hospitalization, institutionalization, and mortality [1]. A major component of frailty is sarcopenia, the progressive loss of muscle mass, strength, and physical function [2].

International Clinical Practice Guidelines for Sarcopenia recommend protein supplementation or a protein-rich diet as part of the management of sarcopenia [2]. However, the relationship between protein intake and frailty is inconsistent. The prevalence of frailty was inversely associated with protein intake in older Italians [3], Japanese [4], American women [5], and Spanish individuals [6]. However, no association between the prevalence of frailty and intake of protein was evident in older British women [7], Koreans [8], and Americans [[9], [10], [11]]. In addition, the prevalence of frailty was inversely associated with animal or plant protein in older Japanese women [12] and Spanish individuals [6], but not in older Americans [9]. Previous meta-analyses comprising four cross-sectional studies reported that higher protein intake was negatively associated with frailty status in older adults [13]. However, a more recent meta-analysis indicated that protein intake—whether absolute, energy-adjusted, or relative to total energy intake—was not significantly associated with frailty in older adults [14]. This analysis also revealed that frail older adults consumed significantly less animal-derived but not plant-based protein than their robust counterparts. These discrepancies between risk of frailty and protein intake could be partly due to the energy intake, as energy intake itself was inversely associated with frailty in older Italian [3], British [7], Korean [8], and Japanese [15] populations. In addition, frailty and intake of protein was inversely associated with adequate energy intake [[3], [4], [5], [6]], but not with inadequate energy intake [[7], [8], [9], [10]].

Regarding the food source, protein from dairy products was not associated with the incidence of frailty in older Americans [11]. Additionally, the intake of dairy products or eggs were not associated with the prevalence, incidence, or transition of frailty in older French [16], Chinese [17], and Italian [18] populations. Conversely, intake of meat, seafood, legumes, and nuts were inversely associated with the prevalence of frailty in older Asian [17,19,20] and American [21] populations. Except for protein from dairy products, the effect of protein intake from food sources on frailty remains unknown.

The present study aimed to investigate the hypothesis that the prevalence of frailty is inversely associated with intake of total, animal, and plant proteins for individuals with sufficient energy intake, but not in those with deficient energy intake. In addition, the association between the prevalence of frailty and various dietary sources of protein was determined.

## 2 Material and methods

### 2.1 Participants

**Fig. 1:**
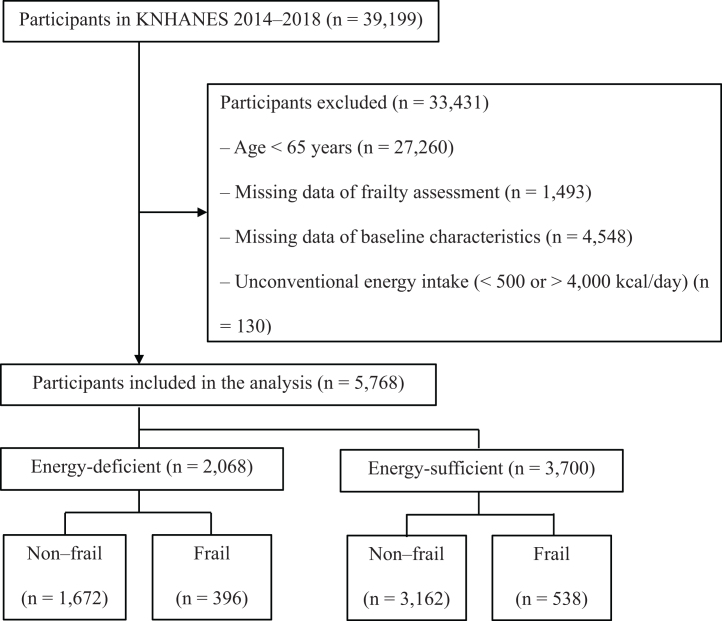
Flowchart of the inclusion and exclusion of participants. KNHANES, Korea National Health and Nutrition Examination Survey.

This study was based on data from the Korea National Health and Nutrition Examination Survey (KNHANES) from 2014 to 2018 conducted by the Korea Disease Control and Prevention Agency. The KNHANES is a cross-sectional nationwide survey using a stratified, multistage, probability sampling design, comprising a health interview, health examination, and nutrition survey [22]. This study was approved by the Hanyang University Institutional Review Board (HYUIRB-202410-024). Of 39,199 participants, 5,768 were included in the study. Participants aged <65 years (n = 27,260), those with missing data of frailty assessment (n = 1,493), missing data of baseline characteristics (n = 4,548), and unconventional energy intake (<500 or >4,000 kcal/day; n = 130) were excluded (Fig. 1). The estimated energy requirement (EER) was calculated using the formula for the Dietary Reference Intakes for Koreans [23]. Daily energy intakes <75% and ≥75% of EER were defined as energy-deficient and energy-sufficient, respectively [23].

### 2.2 Frailty assessment

Frailty was assessed using a modified version of the Cardiovascular Health Study (CHS) frailty index with five criteria: unintentional weight loss, exhaustion, low physical activity, low handgrip strength, and slow walking speed [1]. Participants with a score ≥3 were considered frail. Unintentional weight loss was defined as ≥3 kg loss in the past year. Exhaustion was assessed by the level of stress awareness, and defined by the survey response of ‘I feel considerably exhausted’. Low physical activity was evaluated using the validated Korean version of the International Physical Activity Questionnaire Short-Form, and defined as ≤494.65 kcal/week for men and ≤283.50 kcal/week for women [24]. Low handgrip strength was measured twice or three times for each hand using a model T.K.K. 5401 digital grip strength dynamometer (Takei Scientific Instruments Co. Ltd., Tokyo, Japan), and defined as maximal handgrip strength <28 kg for men and <18 kg for women [25]. Slow walking speed was assessed using the EuroQol-5 Dimension, and defined by the survey response of ‘I have moderate difficulty walking’ or ‘I have to stay in bed all day’.

### 2.3 Study variables

A health interview collected data on age, sex, smoking status, alcohol consumption, education level, economic status, living status, and comorbidities. Economic status referred to National Basic Livelihood Security System recipients. Comorbidities were determined by the presence of ≥2 of hypertension, diabetes mellitus, cancer, chronic obstructive pulmonary disease, myocardial infarction, heart failure, angina, asthma, arthritis, cerebral ischemia, renal disease, or depression. A health examination survey measured height and weight. Body mass index (BMI) was calculated as weight (kg) divided by height squared (m^2^).

Dietary intake was collected using one-day 24-h dietary recall during the household interviews. Energy and protein intake were calculated based on the Food Composition Table from the Korea Rural Development Administration [26]. Protein was classified as plant or animal protein according to dietary sources. Protein from grains, legumes, nuts, seeds, fruits, and vegetables was included in plant protein, while protein from meat, seafood, eggs, and dairy products was included in animal protein. For the analysis, dietary protein intake was categorized into tertiles (T1, T2, and T3). However, due to low consumption, dairy products and eggs were not grouped into tertiles, as 62–67% of participants did not consume these products. Instead, these food groups were combined for analysis. Similarly, the average protein intake from legumes, nuts, and seeds ranged from 0.9 to 4.9 g, which was too low for tertile grouping, particularly among energy-deficient participants. Therefore, these food groups were also combined for analysis.

### 2.4 Statistical analyses

Statistical analyses were performed using the SPSS software (version 27.0; SPSS Inc., Chicago, IL, USA). All analysis was performed using clustering and stratifying variables, using a survey procedure that applied individual weights to the analysis [27]. Continuous variables were analyzed using the independent t-test, and are presented as mean ± standard error of the mean. Categorical variables were analyzed using the chi-square test, and are presented as frequencies and percentages.

In the multivariate models, covariates with p < 0.20 were chosen as confounding factors and included in the adjusted model. Participants were grouped into tertiles based on their dietary protein intake, with T1 representing the lowest intake and the reference group. The association between the prevalence of frailty and protein intake was calculated with odds ratios (ORs) and 95% confidence intervals (CIs) using multivariable logistic regression analysis. The p-value for trend was calculated using the median values within each tertile of dietary intake; p < 0.05 was considered statistically significant.

## 3 Results

The prevalence of frailty was 14.1% and 18.6% in energy-sufficient and deficient participants, respectively, and was significantly lower in energy-sufficient participants (p < 0.001). Frail participants were older and had lower education level, economic status, intake of energy, total, animal, and plant proteins; however a higher proportion of frail participants were women, those living alone, and those with comorbidities than non-frail participants in both energy-sufficient and energy-deficient participants (Table 1). Additionally, frail participants had a lower BMI than non-frail participants in energy-sufficient, but not in energy-deficient participants.

**Table 1:** Baseline characteristics of the frail and non-frail participants in energy-sufficient and energy-deficient participants.

	Energy-sufficient participants (n = 3,700)		*p*-value*	Energy-deficient participants (n = 2,068)		*p*-value*
	Non-frail (n = 3,162)	Frail (n = 538)		Non-frail (n = 1,672)	Frail (n = 396)	
Women, n (%)	1,557 (47.6)	373 (69.0)	<0.001	951 (55.3)	278 (69.0)	<0.001
Age, n (%)			<0.001			<0.001
≤70 years	1,460 (46.4)	124 (23.5)		640 (39.3)	74 (20.5)	
≥71 years	1,702 (53.6)	414 (76.5)		1,032 (60.7)	322 (79.5)	
Body mass index, kg/m^2^	23.92 ± 0.06	23.38 ± 0.19	0.005	24.67 ± 0.09	24.26 ± 0.22	0.085
Current smoker, n (%)	283 (9.0)	62 (10.8)	0.255	148 (9.5)	30 (8.2)	0.459
Current drinker, n (%)	1,839 (59.0)	214 (42.2)	<0.001	810 (49.7)	124 (31.4)	<0.001
Education level, n (%)			<0.001			<0.001
≤Elementary school	1,387 (41.8)	400 (73.8)		899 (51.0)	296 (74.3)	
Middle school	621 (19.2)	68 (12.4)		330 (20.2)	49 (12.1)	
High school	681 (22.4)	49 (8.7)		281 (18.0)	33 (8.1)	
≥College	473 (16.6)	21 (5.1)		162 (10.7)	18 (5.5)	
Low economic status, n (%)	228 (6.3)	81 (13.3)	<0.001	198 (10.8)	85 (19.0)	<0.001
Living alone, n (%)	593 (15.5)	149 (22.2)	<0.001	384 (18.4)	130 (27.5)	<0.001
Comorbidities^a^, n (%)	1,179 (37.1)	286 (53.9)	<0.001	778 (45.4)	230 (57.3)	<0.001
Dietary intake						
Energy intake, kcal	2,056 ± 12.1	1,825 ± 26.4	<0.001	1,130 ± 7.42	996.9 ± 15.0	<0.001
Total protein, g	68.75 ± 0.57	55.90 ± 1.20	<0.001	37.19 ± 0.39	31.54 ± 0.95	<0.001
Animal protein, g	26.40 ± 0.48	18.61 ± 0.95	<0.001	12.66 ± 0.35	10.59 ± 0.88	0.027
Plant protein, g	42.35 ± 0.33	37.30 ± 0.66	<0.001	24.52 ± 0.24	20.95 ± 0.46	<0.001

Data are presented as mean ± standard error of the mean or number of the participants (percentage distribution), as appropriate.

* *p*-values were analyzed using the independent t-test for parametric continuous variables and chi-squared test for categorical variables.

a Comorbidities were determined by the presence of two or more of the following diseases: hypertension, diabetes mellitus, cancer, chronic obstructive pulmonary disease, myocardial infarction, heart failure, angina, asthma, arthritis, cerebral ischemic, renal disease, or depression.

Intake of total, animal, and plant proteins were inversely associated with the prevalence of frailty in energy-sufficient participants (Table 2). In energy-deficient participants, only intake of plant protein from fruits/vegetables was inversely associated with the prevalence of frailty. Intake of animal protein from meat and seafood, and plant protein from legumes/nuts/seeds and fruits/vegetables were also inversely associated with the prevalence of frailty in energy-sufficient participants (Table 3).

**Table 2:** Associations between the daily intake of protein and prevalence of frailty in energy-sufficient and energy-deficient participants.

	Tertiles of dietary intake			*p* for trend
	T1	T2	T3	
Energy-sufficient participants				
Total protein, g	≤51.83	51.83 < to ≤72.54	>72.54	
Frail/non-frail, n	282/951	151/1,083	105/1,128	
OR (95% CI)	1	0.824 (0.630–1.079)	0.668 (0.462–0.967)	0.032
Animal protein, g	≤12.38	12.38 < to ≤27.94	>27.94	
Frail/non-frail, n	254/980	170/1,063	114/1,119	
OR (95% CI)	1	0.820 (0.624–1.078)	0.592 (0.428–0.817)	0.001
Plant protein, g	≤33.41	33.41 < to ≤45.14	>45.14	
Frail/non-frail, n	250/984	158/1,075	130/1,103	
OR (95% CI)	1	0.680 (0.503–0.918)	0.678 (0.462–0.995)	0.043
Energy-deficient participants				
Total protein, g	≤27.97	27.97 < to ≤39.22	>39.22	
Frail/non-frail, n	192/497	124/567	80/609	
OR (95% CI)	1	0.942 (0.674–1.315)	0.808 (0.529–1.234)	0.320
Animal protein, g	≤4.50	4.50 < to ≤13.74	>13.74	
Frail/non-frail, n	174/515	118/572	104/586	
OR (95% CI)	1	0.714 (0.516–0.988)	0.720 (0.487–1.065)	0.133
Plant protein, g	≤19.71	19.71 < to ≤26.30	>26.30	
Frail/non-frail, n	195/495	125/565	76/613	
OR (95% CI)	1	0.707 (0.496–1.008)	0.574 (0.372–0.884)	0.012

OR, odds ratio; CI, Confidence interval. Estimate of *p* for linear trends was based on linear scores derived from the medians of tertiles of protein intake among all participants. Adjusted OR and 95% CI were analyzed using logistic regression analysis after adjusting for sex, age, body mass index, alcohol consumption status, education, low economic status, living alone, comorbidities, and energy intake. Animal protein was adjusted for plant protein and vice versa.

**Table 3:** Associations between the daily intake of protein from dietary sources and the prevalence of frailty in energy-sufficient and energy-deficient participants.

	Tertiles of dietary intake			*p* for trend
	T1	T2	T3	
Energy-sufficient participants				
Meat, g	0	0 < to ≤11.01	>11.01	
Frail/non-frail, n	248/1,084	155/980	135/1,098	
OR (95% CI)	1	0.750 (0.572–0.982)	0.679 (0.503–0.918)	0.024
Seafood, g	≤1.17	1.17 < to ≤7.90	>7.90	
Frail/non-frail, n	233/1,000	187/1,047	118/1,115	
OR (95% CI)	1	0.890 (0.683–1.159)	0.678 (0.503–0.914)	0.011
Dairy products and egg, g	0	0 < to ≤5.91	>5.91	
Frail/non-frail, n	285/1,205	119/858	134/1,099	
OR (95% CI)	1	0.675 (0.502–0.906)	0.801 (0.596–1.077)	0.316
Grain, g	≤17.67	17.67 < to ≤24.78	>24.78	
Frail/non-frail, n	177/1,056	197/1,036	164/1,070	
OR (95% CI)	1	1.080 (0.814–1.433)	1.079 (0.738–1.576)	0.699
Legumes, nuts, and seeds, g	≤2.04	2.04 < to ≤7.21	>7.21	
Frail/non-frail, n	235/999	169/1,063	134/1,100	
OR (95% CI)	1	0.919 (0.696–1.215)	0.713 (0.541–0.940)	0.015
Fruits and vegetables, g	≤4.27	4.27 < to ≤7.55	>7.55	
Frail/non-frail, n	263/971	162/1,071	113/1,120	
OR (95% CI)	1	0.795 (0.593–1.065)	0.599 (0.441–0.815)	0.001
Energy-deficient participants				
Meat, g	0	0 < to ≤3.30	>3.30	
Frail/non-frail, n	237/854	46/243	113/576	
OR (95% CI)	1	0.681 (0.439–1.058)	0.763 (0.545–1.069)	0.178
Seafood, g	≤0.32	0.32 < to ≤3.63	>3.63	
Frail/non-frail, n	158/531	133/558	105/584	
OR (95% CI)	1	0.937 (0.677–1.298)	0.946 (0.666–1.346)	0.836
Dairy products and egg, g	0	0 < to ≤2.07	>2.07	
Frail/non-frail, n	236/851	53/240	107/582	
OR (95% CI)	1	0.800 (0.531–1.205)	0.725 (0.532–0.988)	0.057
Grains, g	≤10.51	10.51 < to ≤14.52	>14.52	
Frail/non-frail, n	161/528	116/575	119/570	
OR (95% CI)	1	0.929 (0.656–1.316)	1.460 (0.954–2.234)	0.075
Legumes, nuts, and seeds, g	≤0.74	0.74 < to ≤3.85	>3.85	
Frail/non-frail, n	169/521	119/571	108/581	
OR (95% CI)	1	0.677 (0.491–0.931)	0.709 (0.517–0.973)	0.093
Fruits and vegetables, g	≤3.00	3.00 < to ≤5.39	>5.39	
Frail/non-frail, n	200/490	119/569	77/613	
OR (95% CI)	1	0.665 (0.481–0.919)	0.400 (0.280–0.571)	<0.001

OR, odds ratio; CI, Confidence interval. Estimate of *p* for linear trends was based on linear scores derived from the medians of tertiles of protein intake among all participants. Adjusted OR and 95% CI were analyzed using logistic regression analysis after adjusting for sex, age, body mass index, alcohol consumption status, education, low economic status, living alone, comorbidities, and energy intake. Animal protein was adjusted for plant protein and vice versa.

Regarding the five frailty criteria, the prevalence of exhaustion and low physical activity were inversely associated with the intake of total and animal protein in energy-sufficient participants (Table 4). The prevalence of low physical activity was inversely associated with the intake of plant protein in energy-deficient participants.

**Table 4:** Associations between the daily intake of protein and the risk of frailty criterion in energy-sufficient and energy-deficient participants.

	Tertiles of dietary intake			*p* for trend
	T1	T2	T3	
Energy-sufficient participants				
Total protein, g	≤51.83	51.83 < to ≤72.54	>72.54	
Weight loss, n (yes/no)	130/1,103	107/1,127	83/1,150	
OR (95% CI)	1	1.250 (0.886–1.762)	1.066 (0.720–1.577)	0.810
Exhaustion, n (yes/no)	270/963	179/1,055	163/1,070	
OR (95% CI)	1	0.791 (0.612–1.023)	0.637 (0.449–0.903)	0.013
Low PA, n (yes/no)	624/609	520/714	434/799	
OR (95% CI)	1	0.772 (0.625–0.954)	0.674 (0.518–0.876)	0.005
Low HGS, n (yes/no)	406/827	251/983	190/1,043	
OR (95% CI)	1	0.820 (0.649–1.036)	0.869 (0.618–1.222)	0.449
Slow WS, n (yes/no)	503/730	391/843	336/897	
OR (95% CI)	1	0.944 (0.760–1.174)	0.987 (0.750–1.299)	0.962
Animal protein, g	≤12.38	12.38 < to ≤27.94	>27.94	
Weight loss, n (yes/no)	134/1,100	97/1,136	89/1,144	
OR (95% CI)	1	0.911 (0.658–1.262)	0.936 (0.640–1.369)	0.767
Exhaustion, n (yes/no)	249/985	193/1,040	170/1,063	
OR (95% CI)	1	0.793 (0.603–1.042)	0.626 (0.463–0.845)	0.003
Low PA, n (yes/no)	596/638	528/705	454/779	
OR (95% CI)	1	0.910 (0.740–1.120)	0.767 (0.615–0.957)	0.016
Low HGS, n (yes/no)	372/862	271/962	204/1029	
OR (95% CI)	1	0.872 (0.695–1.094)	0.837 (0.627–1.119)	0.253
Slow WS, n (yes/no)	481/753	381/852	368/865	
OR (95% CI)	1	0.872 (0.706–1.077)	1.061 (0.830–1.356)	0.508
Plant protein, g	≤33.41	33.41 < to ≤45.14	>45.14	
Weight loss, n (yes/no)	122/1,112	99/1,134	99/1,134	
OR (95% CI)	1	1.206 (0.826–1.761)	1.203 (0.799–1.812)	0.378
Exhaustion, n (yes/no)	245/989	191/1,042	176/1,057	
OR (95% CI)	1	0.826 (0.636–1.076)	0.735 (0.527–1.024)	0.063
Low PA, n (yes/no)	582/652	522/711	474/759	
OR (95% CI)	1	0.813 (0.666–0.992)	0.754 (0.577–0.986)	0.059
Low HGS, n (yes/no)	368/866	263/970	216/1,017	
OR (95% CI)	1	0.693 (0.533–0.902)	0.759 (0.554–1.039)	0.089
Slow WS, n (yes/no)	467/767	413/820	350/883	
OR (95% CI)	1	1.021 (0.815–1.278)	0.958 (0.733–1.254)	0.746
Energy-deficient participants				
Total protein, g	≤27.96	27.96 < to ≤39.22	>39.22	
Weight loss, n (yes/no)	76/613	70/621	58/631	
OR (95% CI)	1	1.084 (0.717–1.636)	1.376 (0.801–2.361)	0.241
Exhaustion, n (yes/no)	155/534	125/566	99/590	
OR (95% CI)	1	1.061 (0.760–1.481)	1.085 (0.713–1.650)	0.718
Low PA, n (yes/no)	361/328	314/377	276/413	
OR (95% CI)	1	0.805 (0.605–1.073)	0.726 (0.516–1.023)	0.082
Low HGS, n (yes/no)	279/410	186/505	145/544	
OR (95% CI)	1	0.868 (0.642–1.174)	0.809 (0.557–1.174)	0.282
Slow WS, n (yes/no)	346/343	275/416	224/465	
OR (95% CI)	1	0.985 (0.733–1.323)	1.072 (0.774–1.485)	0.635
Animal protein, g	≤4.49	4.49 < to ≤13.74	>13.74	
Weight loss, n (yes/no)	80/609	63/627	61/629	
OR (95% CI)	1	0.890 (0.596–1.330)	0.793 (0.489–1.284)	0.354
Exhaustion, n (yes/no)	155/534	108/582	116/574	
OR (95% CI)	1	0.571 (0.416–0.784)	0.907 (0.624–1.319)	0.945
Low PA, n (yes/no)	352/337	299/891	300/390	
OR (95% CI)	1	0.749 (0.577–0.971)	0.779 (0.568–1.069)	0.199
Low HGS, n (yes/no)	264/425	171/519	175/515	
OR (95% CI)	1	0.630 (0.473–0.838)	0.837 (0.604–1.160)	0.492
Slow WS, n (yes/no)	313/376	296/394	236/454	
OR (95% CI)	1	1.067 (0.815–1.398)	1.104 (0.815–1.496)	0.546
Plant protein, g	≤19.71	19.71 < to ≤26.30	>26.30	
Weight loss, n (yes/no)	86/604	53/637	65/624	
OR (95% CI)	1	0.647 (0.402–1.043)	1.137 (0.637–2.029)	0.590
Exhaustion, n (yes/no)	152/538	129/561	98/591	
OR (95% CI)	1	1.101 (0.776–1.561)	1.084 (0.695–1.692)	0.734
Low PA, n (yes/no)	359/331	327/363	265/424	
OR (95% CI)	1	0.726 (0.557–0.947)	0.472 (0.337–0.651)	<0.001
Low HGS, n (yes/no)	273/417	201/489	136/553	
OR (95% CI)	1	0.837 (0.616–1.137)	0.708 (0.475–1.056)	0.092
Slow WS, n (yes/no)	334/356	292/398	219/470	
OR (95% CI)	1	0.938 (0.704–1.249)	0.730 (0.518–1.027)	0.062

OR, odds ratio; CI, Confidence interval. Estimate of *p* for linear trends was based on linear scores derived from the medians of tertiles of protein intake among all participants. Adjusted OR and 95% CI were analyzed using logistic regression analysis after adjusting for sex, age, body mass index, drinking status, education, low economic status, living alone, comorbidities, and energy intake. Animal protein was adjusted for plant protein and vice versa. PA, physical activity; HGS, handgrip strength; WS, walking speed.

Baseline characteristics and associations between protein intake, prevalence of frailty, and frailty criteria were similar in the total and energy-sufficient participants (Supplemental Tables S1–S4). The prevalence of low handgrip strength was inversely associated with the intake of total and plant proteins in all participants. The prevalence of low physical activity was also inversely associated with the intake of plant protein in all participants.

## 4 Discussion

In this study, total and animal protein intake were inversely associated with the prevalence of frailty among energy-sufficient participants, whereas plant protein intake was associated with a lower frailty prevalence, regardless of energy intake. These findings suggest that the relationship between protein intake and the prevalence of frailty may be influenced by overall energy intake and highlight the potential role of both protein source and quantity in preventing frailty. Previous studies reported inverse associations between the prevalence or incidence of frailty and protein intake in older Italians [3], Japanese [4], American women [5], and Spanish individuals [6]. The approximate average intake of energy of 1,737–2,037 kcal [[3], [4], [5], [6]] are adequate according to the Dietary referenc intakes (DRIs) of their respective countries (1,650–2,400 kcal) [[28], [29], [30]]. Conversely, the prevalence or incidence of frailty was not associated with protein intake in older British women [7], Koreans [8], and Americans [9,10]. The approximate average intakes of energy (1,243–1,833 kcal) were lower than the DRIs of their respective countries (1500–2,300 kcal) [23,31,32]. Energy intake has been consistently inversely associated with the prevalence of frailty in older Italians [3], British [7], Korean [8], and Japanese [15] populations. In a controlled diet intervention trial, consumption of 1.5 g/kg protein with an energy-deficient diet (80% of EER) reduced muscle protein synthesis by decreased phosphorylation of intracellular signaling proteins compared to the consumption of 1.5 g/kg protein with 100% of EER in young American adults [33]. In the present study, average energy intake was 2,014 and 1,098 kcal in energy-sufficient and deficient participants, respectively. The energy DRIs of the older Koreans was 1,677–1,783 kcal depending on the age and sex, showing that the energy-sufficient participants received sufficient energy [23]. Thus, the prevalence of frailty was inversely associated with protein intake in energy-sufficient, but not energy-deficient, participants.

A meta-analysis of three cross-sectional studies found that frail older adults consumed significantly less animal protein than their robust counterparts [14]. Furthermore, animal protein intake was inversely associated with the prevalence and incidence of frailty in older Japanese women [12] and Spanish individuals [6], but not in Americans [9]. This inconsistency could be attributed to differences in energy intake. Frailty was inversely associated with the intake of animal protein in populations with adequate energy intake according to the DRIs of their countries [6,12], but not in a population with lower energy intake than the DRIs [9]. Consistently, we observed that the intake of animal protein was inversely associated with frailty prevalence in energy-sufficient, but not deficient, participants, suggesting that the potential benefits of animal proteins in reducing frailty risk may depend on adequate energy intake. Sufficient energy intake is crucial for optimizing the utilization of dietary protein for muscle maintenance and function [34]. When energy intake is insufficient, dietary protein may be used as an energy source rather than for muscle preservation, diminishing its protective effect against frailty.

Struijk et al. [11] reported that protein from dairy products was not associated with the incidence of frailty in older Americans. The prevalence or incidence of frailty was not associated with the intake frequency of dairy products and eggs in older French [16] and Chinese [17] individuals, respectively. No significant association of protein intake from dairy products and eggs with the prevalence of frailty was evident. The prevalence or incidence of frailty has been inversely associated with intake frequency of meat in older Chinese [17] and Japanese [18] individuals, and with seafood intake in older Koreans [20]. In the present study, intakes of protein from meat and seafood were inversely associated with the prevalence of frailty among energy-sufficient, but not among energy-deficient participants. The average daily intake of meat was reportedly 51–59 g in older Japanese [35] and Chinese [36] individuals, and that of seafood was 48 g in older Koreans [20]; these values are similar to the 61 g of meat and 52 g of seafood in energy-sufficient participants in the present study. However, the average intake of meat and seafood was 24 g and 28 g in energy-deficient participants, respectively, lower than previous values.

Intake of plant protein was inversely associated with the prevalence or incidence of frailty in older Japanese women [12] and Spanish individuals [6] whose energy intake was adequate, but not in older Americans with an inadequate intake of energy [9]. In the present study, the association was similar to that of previous studies among energy-sufficient, but not deficient, participants. The intake of plant protein in our energy-deficient participants was 67% of the total protein, compared to 41% in the previous study, with no significant association observed [9]. Legumes are recognized as a high-quality plant protein source owing to their relatively similar digestibility and essential amino acid profile to animal proteins [37]. Intake of protein from legumes was 9% of total protein in energy-deficient participants, but 2% in older Americans [38].

The prevalence or incidence of frailty was inversely associated with the intake frequency of legumes in older Chinese [17] and with nuts in older Chinese [17] and American [21] individuals. Intake of protein from legumes/nuts/seeds was inversely associated with the prevalence of frailty in energy-sufficient, but not deficient, participants in the present study. The reported association of prevalence or incidence of frailty with consumption of ≥4 g of nuts [21] is similar to the 6 g value in our energy sufficient participants; however, our energy-deficient participants consumed only 3 g. Intake of fruits and vegetables was inversely associated with the prevalence or incidence of frailty in older Europeans [39,40]. Consistently, there was inverse association between fruit and vegetable and prevalence of frailty in all participants in the present study. Conversely and consistent with the present study, Tanaka et al. [41] reported that grain intake was not correlated with the prevalence of frailty in older Americans.

Regarding frailty criteria, exhaustion was not associated with intake of total and animal proteins in older Spanish [6] and American [9] populations, where exhaustion was assessed using a question from the Center for Epidemiological Studies Depression scale. Conversely, in this study the intake of total and animal proteins were inversely associated with exhaustion evaluated through stress awareness. Physical activity was positively associated with intake of protein, but not with the intake of animal protein in older Americans [9] and not with the intake of total and animal proteins in older Spanish individuals [6]. In the present study, physical activity was positively associated with total and animal protein intake in energy-sufficient participants; moreover, average daily intake of protein was similar to older Americans [9], but not older Spanish individuals [6]. However, average daily intake of animal protein in energy-sufficient participants was not similar to both older American [9] and Spanish [6] individuals. The discrepancies between studies could be partly due to the intake quantity. Adequate protein intake helps limit and treat age-related declines in muscle mass, strength, and functional abilities [42], and animal-derived protein is positively associated with muscle mass and strength across age [43]. Among energy-deficient participants, protein intake was generally low, and only plant protein intake showed a positive association with physical activity.

This study examined the relationship between protein intake, including animal and plant proteins and frailty, according to energy intake status. However, this study had a few limitations. First, a modified version of the CHS frailty index was used, where exhaustion and low walking speed were self-reported rather than measured. Second, the dietary intake data were obtained using the 24-h dietary recall method, which could have recall bias and did not reflect the participants’ usual diet. Third, although adjustments were made for various confounders, residual confounders were possible. Finally, owing to the cross-sectional study design, this study was unable to identify the causal relationship between protein intake and the prevalence of frailty.

## Conclusions

In conclusion, our findings suggest that higher total and animal protein intake is associated with a lower prevalence of frailty among older adults with adequate energy intake, whereas higher plant protein intake is associated with a lower prevalence of frailty, regardless of energy intake. Further large-population-based longitudinal studies are required to validate the observed associations between protein intake and the incidence of frailty across diverse populations.

## CRediT authorship contribution statement

SK: Methodology, Formal analysis, and Writing – original draft. YJ: Supervision, Writing – review & editing. YP: Conceptualization, Funding acquisition, Supervision, and Writing – review & editing. All authors have read and approved the final version of the manuscript.

## Statement of ethics

This study protocol was reviewed and approved by Hanyang University Institutional Review Board [approval number HYUIRB-202410-024]. Written informed consents to participate in KNHANES were obtained from the participants and the survey collection process was approved by the Korea Disease Control and Prevention Agency Research Ethics Review Committee.

## Funding sources

This study was supported by the BK21 Fostering Outstanding Universities for Research (FOUR) project of the National Research Foundation of Korea Grant, and a National Research Foundation of Korea (NRF) grant funded by the Korean government (MSIT) (grant number NRS RS-2024-00334109). The funder had no role in the design, data collection, data analysis, and reporting of this study.

## Data availability statement

The datasets presented in this study can be found in online repositories. The names of the repository/repositories and accession number(s) can be found below: https://knhanes.kdca.go.kr/knhanes/sub03/sub03_01.do.

## Declaration of competing interest

The authors have no conflicts of interest to declare.
