# Supplementary material for: Impact of energy intake on the association between protein intake and the prevalence of frailty in older Korean adults: The Korea National Health and Nutrition Examination Survey, 2014–2018
Source: J Nutr Health Aging. 2025 Feb 19;29(4):100518. doi: 10.1016/j.jnha.2025.100518 (PMC12180019; doi:10.1016/j.jnha.2025.100518)
Supplement: Supplementary file 1 [file mmc1.docx]

**Supplementary Table 1.** Baseline characteristics of the frail and non-frail participants in the total participants

|  | Total participants (n = 5,768) | | *p*-value^*^ |
| --- | --- | --- | --- |
|  | Non-frail (n = 4,834) | Frail (n = 934) |  |
| Women, n (%) | 2,508 (50.3) | 651 (69.0) | <0.001 |
| Age, n (%) |  |  | <0.001 |
| ≤ 70 years | 2,100 (43.9) | 198 (22.2) |  |
| ≥ 71 years | 2,734 (56.1) | 736 (77.8) |  |
| Body mass index, kg/m^2^ | 24.18 ± 0.05 | 23.75 ± 0.14 | 0.004 |
| Current smoker, n (%) | 431 (9.2) | 92 (9.7) | 0.666 |
| Current drinker, n (%) | 2,649 (55.8) | 338 (37.7) | <0.001 |
| Education level, n (%) |  |  | <0.001 |
| ≤ Elementary school | 2,286 (45.0) | 696 (74.0) |  |
| Middle school | 951 (19.5) | 117 (12.3) |  |
| High school | 962 (20.9) | 82 (8.5) |  |
| ≥ College | 635 (14.6) | 39 (5.3) |  |
| Low economic status, n (%) | 426 (7.9) | 166 (15.7) | <0.001 |
| Living alone, n (%) | 977 (16.5) | 279 (24.5) | <0.001 |
| Comorbidities^a^, n (%) | 1,957 (39.9) | 516 (55.4) | <0.001 |
| Dietary intake |  |  |  |
| Energy intake, kcal | 1,736 ± 12.6 | 1,474 ± 23.1 | <0.001 |
| Total protein, g | 57.84 ± 0.51 | 45.58 ± 0.93 | <0.001 |
| Animal protein, g | 21.65 ± 0.37 | 15.21 ± 0.68 | <0.001 |
| Plant protein, g | 36.19 ± 0.30 | 30.37 ± 0.54 | <0.001 |

Data are presented as mean ± standard error of the mean or number of the participants (percentage distribution), as appropriate. ^*^*p*-values were analyzed using the independent t-test for parametric continuous variables and chi-squared test for categorical variables. ^a^Comorbidities were determined by the presence of two or more of the following diseases: hypertension, diabetes mellitus, cancer, chronic obstructive pulmonary disease, myocardial infarction, heart failure, angina, asthma, arthritis, cerebral ischemic, renal disease, or depression.

**Supplementary Table 2**. Associations between the daily intake of protein and the prevalence of frailty in the total participants

|  | Tertiles of dietary intake | | | *p* for trend |
| --- | --- | --- | --- | --- |
|  | T1 | T2 | T3 |  |
| **Total participants** |  |  |  |  |
| Total protein, g | ≤ 40.29 | 40.29 < to ≤ 61.71 | > 61.71 |  |
| Frail/non-frail, n | 464/1,459 | 293/1,630 | 177/1,746 |  |
| OR (95% CI) | 1 | 0.763 (0.607–0.958) | 0.619 (0.437–0.878) | 0.007 |
| Animal protein, g | ≤ 8.54 | 8.54 < to ≤ 22.35 | > 22.35 |  |
| Frail/non-frail, n | 436/1,487 | 291/1,632 | 207/1,716 |  |
| OR (95% CI) | 1 | 0.789 (0.641–0.970) | 0.700 (0.533–0.918) | 0.012 |
| Plant protein, g | ≤ 26.67 | 26.67 < to ≤ 39.25 | > 39.25 |  |
| Frail/non-frail, n | 443/1,479 | 293/1,630 | 198/1,726 |  |
| OR (95% CI) | 1 | 0.734 (0.590–0.914) | 0.638 (0.452–0.900) | 0.011 |

OR, odds ratio; CI, Confidence interval. Estimate of *p* for linear trends was based on linear scores derived from the medians of tertiles of protein intake among all participants. Adjusted OR and 95% CI were analyzed using logistic regression analysis after adjusting for sex, age, body mass index, alcohol consumption status, education, low economic status, living alone, comorbidities, and energy intake. Animal protein was adjusted for plant protein and vice versa.

**Supplementary Table 3**. Associations between the daily intakes of protein from food sources and the prevalence of frailty in the total participants

|  | Tertiles of dietary intake | | | *p* for trend |
| --- | --- | --- | --- | --- |
|  | T1 | T2 | T3 |  |
| **Total participants** |  |  |  |  |
| Meat, g | 0 | 0 < to ≤ 7.90 | > 7.90 |  |
| Frail/non-frail, n | 485/1,938 | 205/1,218 | 244/1,679 |  |
| OR (95% CI) | 1 | 0.725 (0.581–0.905) | 0.715 (0.570–0.897) | 0.011 |
| Seafood, g | ≤ 0.74 | 0.74 < to ≤ 6.13 | > 6.13 |  |
| Frail/non-frail, n | 407/1,516 | 316/1,606 | 211/1,713 |  |
| OR (95% CI) | 1 | 0.911 (0.742–1.119) | 0.686 (0.543–0.867) | 0.002 |
| Dairy products and eggs, g | 0 | 0 < to ≤ 4.53 | > 4.53 |  |
| Frail/non-frail, n | 521/2,056 | 182/1,087 | 231/1,692 |  |
| OR (95% CI) | 1 | 0.730 (0.579–0.922) | 0.763 (0.610–0.955) | 0.050 |
| Grains, g | ≤ 13.99 | 13.99 < to ≤ 21.20 | > 21.20 |  |
| Frail/non-frail, n | 357/1,565 | 305/1,619 | 272/1,651 |  |
| OR (95% CI) | 1 | 1.007 (0.795–1.275) | 1.357 (0.971–1.897) | 0.072 |
| Legumes, nuts, and seeds, g | ≤ 1.48 | 1.48 < to ≤ 5.99 | > 5.99 |  |
| Frail/non-frail, n | 407/1,515 | 283/1,641 | 244/1,679 |  |
| OR (95% CI) | 1 | 0.743(0.599–0.921) | 0.777 (0.630–0.958) | 0.049 |
| Fruits and vegetables, g | ≤ 4.27 | 4.27 < to ≤ 7.55 | > 7.55 |  |
| Frail/non-frail, n | 483/1,439 | 266/1,656 | 185/1,739 |  |
| OR (95% CI) | 1 | 0.596 (0.478–0.743) | 0.483 (0.378–0.618) | <0.001 |

OR, odds ratio; CI, Confidence interval. Estimate of *p* for linear trends was based on linear scores derived from the medians of tertiles of protein intake among all participants. Adjusted OR and 95% CI were analyzed using logistic regression analysis after adjusting for sex, age, body mass index, alcohol consumptn status, education, low economic status, living alone, comorbidities, and energy intake. Animal protein was adjusted for plant protein and vice versa.

**Supplementary Table 4.** Associations between the daily intake of protein and the risk of frailty criterion in the total participants

|  | Tertiles of dietary intake | | | *p* for trend |
| --- | --- | --- | --- | --- |
|  | T1 | T2 | T3 |  |
| **Total participants** |  |  |  |  |
| Total protein, g | ≤ 40.29 | 40.29 < to ≤ 61.71 | > 61.71 |  |
| Weight loss, n (yes/no) | 214/1,709 | 178/1,745 | 132/1,791 |  |
| OR (95% CI) | 1 | 0.944 (0.710–1.254) | 0.925 (0.608–1.408) | 0.726 |
| Exhaustion, n (yes/no) | 405/1,518 | 327/1,596 | 259/1,664 |  |
| OR (95% CI) | 1 | 0.841 (0.682–1.037) | 0.708 (0.518–0.968) | 0.033 |
| Low PA, n (yes/no) | 968/955 | 847/1,076 | 714/1,209 |  |
| OR (95% CI) | 1 | 0.877 (0.737–1.043) | 0.765 (0.609–0.962) | 0.022 |
| Low HGS, n (yes/no) | 676/1,247 | 473/1,450 | 308/1,615 |  |
| OR (95% CI) | 1 | 0.778 (0.640–0.946) | 0.656 (0.492–0.874) | 0.005 |
| Slow WS, n (yes/no) | 862/1,061 | 678/1,245 | 535/1,388 |  |
| OR (95% CI) | 1 | 0.950 (0.792–1.139) | 0.966 (0.750–1.245) | 0.824 |
| Animal protein, g | ≤ 8.53 | 8.53 < to ≤ 22.35 | > 22.35 |  |
| Weight loss, n (yes/no) | 217/1,706 | 166/1,757 | 141/1,782 |  |
| OR (95% CI) | 1 | 0.796 (0.622–1.019) | 0.906 (0.660–1.244) | 0.648 |
| Exhaustion, n (yes/no) | 402/1,521 | 314/1,609 | 275/1,648 |  |
| OR (95% CI) | 1 | 0.799 (0.653–0.976) | 0.728 (0.562–0.943) | 0.023 |
| Low PA, n (yes/no) | 945/978 | 841/1,082 | 743/1,180 |  |
| OR (95% CI) | 1 | 0.906 (0.768–1.070) | 0.807 (0.663–0.983) | 0.035 |
| Low HGS, n (yes/no) | 625/1,298 | 487/1,436 | 345/1,578 |  |
| OR (95% CI) | 1 | 0.963 (0.810–1.143) | 0.807 (0.645–1.011) | 0.057 |
| Slow WS, n (yes/no) | 826/1,097 | 668/1,255 | 581/1,342 |  |
| OR (95% CI) | 1 | 0.937 (0.793–1.108) | 1.032 (0.848–1.255) | 0.658 |
| Plant protein, g | ≤ 26.67 | 26.67 < to ≤ 39.25 | > 39.25 |  |
| Weight loss, n (yes/no) | 197/1,725 | 183/1,740 | 144/1,780 |  |
| OR (95% CI) | 1 | 1.114 (0.846–1.468) | 1.159 (0.775–1.732) | 0.490 |
| Exhaustion, n (yes/no) | 395/1,527 | 326/1,597 | 270/1,654 |  |
| OR (95% CI) | 1 | 0.874 (0.707–1.079) | 0.747 (0.553–1.009) | 0.059 |
| Low PA, n (yes/no) | 941/981 | 826/1,097 | 762/1,162 |  |
| OR (95% CI) | 1 | 0.775 (0.654–0.917) | 0.767 (0.606–0.971) | 0.046 |
| Low HGS, n (yes/no) | 625/1,298 | 487/1,436 | 345/1,578 |  |
| OR (95% CI) | 1 | 0.751 (0.613–0.919) | 0.685 (0.512–0.916) | 0.014 |
| Slow WS, n (yes/no) | 855/1,067 | 644/1,279 | 576/1,348 |  |
| OR (95% CI) | 1 | 0.807 (0.678–0.960) | 0.971 (0.762–1.239) | 0.992 |

OR, odds ratio; CI, Confidence interval. Estimate of *p* for linear trends was based on linear scores derived from the medians of tertiles of protein intake among all participants. Adjusted OR and 95% CI were analyzed using logistic regression analysis after adjusting for sex, age, body mass index, alcohol consumption status, education, low economic status, living alone, comorbidities, and energy intake. Animal protein was adjusted for plant protein and vice versa. PA, physical activity; HGS, handgrip strength; WS, walking speed
